# Regression-based gap-filling methods show air temperature reductions and wind pattern changes during the 2019 total eclipse in Chile

**DOI:** 10.1038/s41598-022-10623-z

**Published:** 2022-05-11

**Authors:** Arno C. Hammann, Shelley MacDonell

**Affiliations:** 1 Asiaq - Greenland Survey, Qatserisut 8, Nuuk, Greenland; 2 Centro de Estudios Avanzados en Zonas Áridas, Raúl Bitrán 1305, La Serena, Chile; 3 Waterways Centre for Freshwater Management, University of Canterbury and Lincoln University, Private Bag 4800, Christchurch, New Zealand

**Keywords:** Atmospheric dynamics, Statistics, Scientific data

## Abstract

Singular disruptive events like solar eclipses affect the measured values of meteorological variables at the earth’s surface. To quantify such an impact, it is necessary to estimate what value the parameter would have taken had the event not occurred. We design and compare several methods to perform such an estimate based on longer observational timeseries from individual meteorological surface stations. Our methods are based on regularised regressions (including a Bayesian variant) and provide both a point an associated error estimate of the disruptive event’s impact. With their help, we study the effect of the total solar eclipse of July 2nd, 2019, in the Coquimbo Region of Chile, on near-surface air temperatures and winds. The observational data used have been collected by the meteorological surface station network of the Centro de Estudios Avanzados en Zonas Áridas (CEAZA). Most stations inside the eclipse’s umbra registered a temperature drop of 1–2 $^{\circ }$C, while the most extreme estimated temperature drop surpassed 6 $^{\circ }$C. The presence of an ‘eclipse cyclone’ can neither be proven nor refuted. Application of the regression methods to other comparable problems like volcanic eruptions, forest fires, or simply gap filling of observational data, are conceivable.

## Introduction

Certain singular events, such as solar eclipses, volcanic eruptions or forest fires, cause a disruption to the solar radiation receipt at the earth’s surface and throughout the atmospheric column. Other meteorological variables are affected as a result; for example, surface air temperatures may experience a temporary drop. In order to determine how much of a temperature drop (or corresponding perturbations in other variables) is caused directly by the singular event, it is necessary to estimate what the variable’s value would have been in its absence. In this study, we apply and compare a number of statistical techniques to the problem of estimating the effect of a specific solar eclipse on near-surface air temperatures and winds, with a particular focus on the associated uncertainties (an aspect almost universally neglected in existing studies). The methods we explore comprise several types of regularised regressions which have not been applied to this problem before, together with some approaches which have previously been described in the literature but whose uncertainties have not been evaluated. While changes in surface air temperatures are commonly reported as a result of solar eclipses, a certain lore has also accumulated around the idea of an eclipse wind^1,2^, and the particular theory of the eclipse-induced cyclone^3^ has attracted various attempts at proving or refuting it^4,5^. Due to the fluctuating nature of wind directions, estimating eclipse-induced changes is particularly challenging. We apply the best-performing method from our tests with temperature to the problem of estimating the eclipse wind. While the methods can be applied to other variables, the quality of our records is best for temperatures and winds, which are also widely reported by previous studies (facilitating comparisons). Other applications to short-duration singular events are conceivable; we want to emphasize that longer-term changes (including climate change) cannot be addressed or would require a retooling of the methods. The specific eclipse during which we collected data occurred on July 2nd, 2019, in the Coquimbo Region of Chile, approximately 400 km north of the capital Santiago; it was an eclipse of Saros cycle 127.

Quantitative accounts of the meteorological effects of solar eclipses can be found in the literature as early as 1834^1^, and include records of observations from the earth’s surface layer, the stratosphere^6^ and the ionosphere^7^. Many restrict themselves to documenting the evolution of measured quantities over the course of the eclipse, especially if instrumentation has been put in place only for the event itself^6,8–14^. Attempts to diagnose what part of the system’s evolution is due to the direct impact of an eclipse have also been made, usually by comparison to its prior or posterior state^15–23^ or to a physics-based model which excludes the eclipse-specific radiative forcing. Both simpler column models of the atmospheric surface layer^24,25^ and complete numerical weather prediction models (NWP) have been used. Studies using NWPs have either compared simulations with and without eclipse-specific radiative forcing^26,27^, or simulations without eclipse forcing and observations made during an eclipse^2,4^.

If a sufficient number of parameters can be measured, they can be causally related through physical models and an eclipse’s impact can be directly described. For the atmospheric surface layer, important variables include radiative and turbulent fluxes of energy, and potentially also advective fluxes and some measure of the boundary layer depth. Under such conditions, the reduction in radiative heating of the earth’s surface can be connected directly to the response of the atmospheric boundary layer^12–14,21,28–32^. Unfortunately, such a complete set of measurements is rarely available (it was not in our case), and the eclipse’s effect needs to be deduced rather than observed. Provided that the variable of interest is observed, the fundamental problem is to obtain an estimate of how this quantity would have evolved in the eclipse’s absence. We call this estimated ‘uneclipsed’ evolution the *reference*. Existing studies frequently use a quantity’s value at or before First Contact as a reference^19,20,26,33,34^. First Contact (‘C1’) denotes the time point when the two discs of sun and moon appear to touch for the first time to an observer at a given geographic location; Fourth Contact (‘C4’) is the time point when the contact between the discs ceases. Second (‘C2’) and Third (‘C3’) Contact bracket totality and are not defined for partial eclipses. Taking into account the likely existence of a lag between radiative forcing and atmospheric response, one might estimate the eclipse’s effect as the difference between a temperature maximum reached shortly after C1 and the minimum (likely reached slightly after maximum occultation), i.e. as the temperature *range* during the eclipse^35^. Another slight variation is to use a short-term mean of values before C1 and after C4^8,36^. Such approaches may be appropriate outside of transition periods like sunrise^16^ and -set, and where the variable of interest does not typically exhibit large fluctuations on short time scales.

Complete days preceding or following the day of an eclipse are another popular choice to obtain reference values^37–40^, and ‘similar’ days in a more general sense have also been used^32,41^. This overcomes the issue with using persistence as estimation principle during rapid transition periods (sunrise/-set) and is useful if little information is available otherwise as to how a quantity (e.g. air pressure^42^, winds^5,40^, ozone^10^, aerosol concentrations^41,43^, radar reflectivity^44^, atmospheric electric potential^45^) typically evolves. An absence of large and rapid fluctuations, however, is nonetheless desirable for estimates based on single reference days to be meaningful. While solar radiation at the earth’s surface is subject to some variability due to short-term variations in aerosol optical depth^46^, the impact of an eclipse is quantitatively much larger and can therefore be estimated fairly accurately (as long as clouds do not complicate the picture)^28,40,47–49^.

If the measured quantity exhibits large intrinsic variability, some form of regularisation of the reference estimate is necessary. Averaging over several reference days can provide such regularisation^29,43,50,51^, as can the smoothing of eclipse-day observations. Linear interpolation of the variable’s values between C1 and C4^31,35^ may be the simplest variant of such smoothing; higher-order polynomials^52^, splines^53^, local regression (under exclusion of observations around the time of the eclipse)^5^ and fits to variable-specific parametric models^9^ have also been used.

Regularisation of the reference estimate was particularly important for the eclipse of July 2nd, 2019, which occurred so close to sunset (Table 1) that many air temperature timeseries do not show a clear rise in temperatures between C4 and sunset, as they typically do if the eclipse occurs in the middle of the day. Additionally, Chile’s Coquimbo region borders the cold coastal waters of the Humboldt current, and slight changes in wind direction can lead to sudden drops in local air temperatures due to the advection of cold air from the marine boundary layer onto land. The magnitude and rate-of-change of such advective temperature changes are indistinguishable from those registered during the eclipse.

**Table 1:** ‘Contact’ times of the eclipse of July 2nd, 2019, calculated for La Serena (29°54$'$16.3$''$ S 71°14$'$56.2$''$ W) on http://xjubier.free.fr/en/ and rounded to the nearest minute, in local time (UTC-4). Also given are the time of sunset (calculated on https://planetcalc.com/) and time ranges referred to in the text: ‘$t_{\bullet }$’ refers to the times removed from statistical calculations to avoid training data containing effects of the eclipse, and ‘$\overline{u}$, $\overline{v}$’ refers to the time span over which wind data is averaged.

C1	15:23
C2	16:38
Max. occultation	16:39
C3	16:40
C4	17:47
Sunset	17:56
$t_{\bullet }$	15:20–19:00
$\overline{u}$, $\overline{v}$	17:00–17:30

## Results

### Terminology

All discussions below apply to timeseries of data from a single sensor, in particular screen level air temperature. We think of such a timeseries as a data matrix $X_{dt}$ indexed by a calendar date $d$ (rows; ‘date axis’) and a time $t$ (columns; ‘time axis’). The time period affected by the eclipse will be given the symbol $t_{\bullet }$; this includes the full duration of the eclipse (C1–C4), plus an additional time span beyond C4 to account for some dynamical lag in the system (see Table 1). Conversely, all times not affected by the eclipse are symbolised by $t_{\circ }$. Analogously, the day of the eclipse will be denoted $d_{\bullet }$ and all other days by $d_{\circ }$. When we talk about an average over a number of days, we mean that the averaging is performed over the date dimension and the result is a one-dimensional array indexed by times (a daily cycle). All data used in this study comes from the meteorological surface station network operated by the Centro de Estudios Avanzados en Zonas Áridas (CEAZA) in La Serena, Chile, from here on called the CEAZAMet station network.

### Evaluation of the regression methods with respect to 2m air temperatures

Our methods are generalisations of two types of estimates found in the literature: (1) averages over a number of ‘similar’ days, and (2) smoothed observations from the day of the eclipse with interpolated values for $t_{\bullet }$^1,2^. Approach (1) can formally be seen as a regression with selected days $d \in d_{\circ }$ serving as predictors and equal regression weights which sum to 1. Dropping the restrictions on the weights and allowing for an intercept generalises the approach, and any regression method with subset selection replaces the task of choosing ‘similar’ days with an objective procedure. Including an intercept can be interpreted as allowing for slowly-varying (synoptic) background conditions. Since we need to judge the similarity of days without considering $t_{\bullet }$, this regression is trained on $t_{\circ }$ even though we are interested in $t_{\bullet }$, for which the model is simply evaluated. Approach (2) can equally be cast as a regression, with $t \in t_{\bullet }$ as targets and $t \in t_{\circ }$ as predictors, trained on examples $d \in d_{\circ }$. This procedure is equivalent to a smoothing along the time axis with the kernel’s weights fit by regression instead of having been determined a priori. We will call the two approaches ‘average’ and ‘smoothing’ type regressions, respectively. In both cases we can make use of the additional information we have from years of monitoring data at the stations at which we observe the eclipse; we use approximately 4 years of data preceding the eclipse, since for this time period, most CEAZAMet stations in the eclipse’s umbra have been collecting data at a 5 min sampling rate.

We use the lasso as a regression method with subset selection (for both the ‘average’ and the ‘smoothing’ type); the predictors are selected by including an $\ell ^1$ penalty on the vector of regression weights during the loss minimization, which effectively sets many of the weights to zero^54^. For comparison, we evaluate unweighted averages over $N$ days similar to the eclipse day (‘N-averages’ from now on), as well as standard smoothing procedures (local regression, in both linear^5^ and quadratic variants). Similarity between predictor and target days for the N-average is assessed as the pairwise Euclidean distance in the space spanned by times $t_{\circ }$. Uncertainties for all methods can be quantified empirically by estimating the values for all $t \in t_{\bullet }$ on all days $d \in d_{\circ }$ and subtracting the corresponding actual observations. This gives an error distribution for each time $t \in t_{\bullet }$. For the smoothing regression—the only method where the quantity of interest to us is actually the target—care needs to be taken to separate training and test data. To this end, we use four-fold cross-validation, so that approximately 1 year of data is in the test set at any time, and we calculate the error for a day only if it is in a test set.

**Figure 1:**
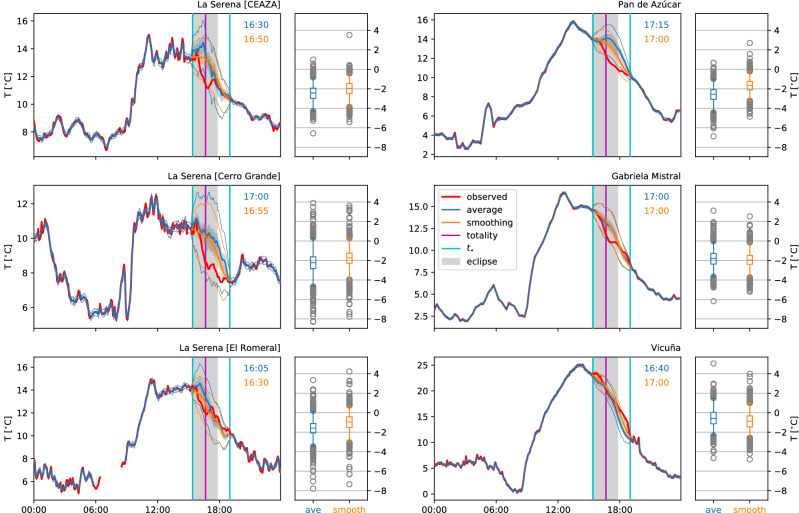
Observed 2m air temperatures on the day of the eclipse (July 2nd, 2019), together with estimates of the ‘uneclipsed’ temperature evolution, for selected stations in the vicinity of La Serena, Chile (left plot of each panel). The reference estimates are obtained from average (‘ave’) and smoothing (‘smooth’) style lasso regressions. Box plots (right) show maximum temperature depression for each estimation method, with corresponding times in the right upper corners of main plot. Boxes (right) and shaded bands (left) correspond to the IQR, whiskers (right) and thin lines (left) to 95% of the data.

**Figure 2:**
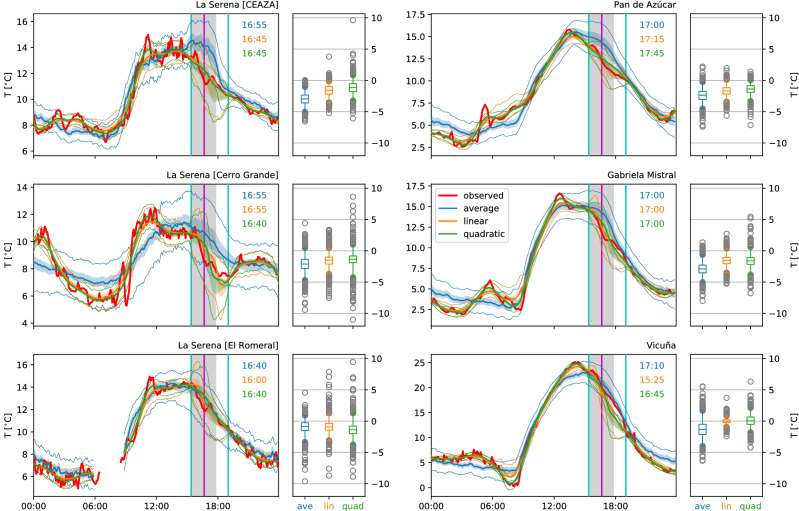
Observed 2m air temperatures and reference estimates as in Fig. 1, but with estimates obtained from unweighted averages over a number of ‘similar’ days and local linear and quadratic regression (a type of smoothing).

Figures 1 and 2 show the regression-based estimates and those obtained from comparison methods, respectively, for air temperature on the day of the eclipse at a few selected stations in and around La Serena. The box plots show the temperature depression $\Delta T$ of the observation relative to the reference estimate. The plot elements representing uncertainty (shaded bands, boxes, whiskers and fliers) are constructed by adding the error distributions computed as described above to the point estimate. The regression methods’ uncertainty is much reduced relative to that of the comparison methods; some but not all estimates of $\Delta T$ fall outside of 95% of the calculated error distributions.

**Figure 3:**
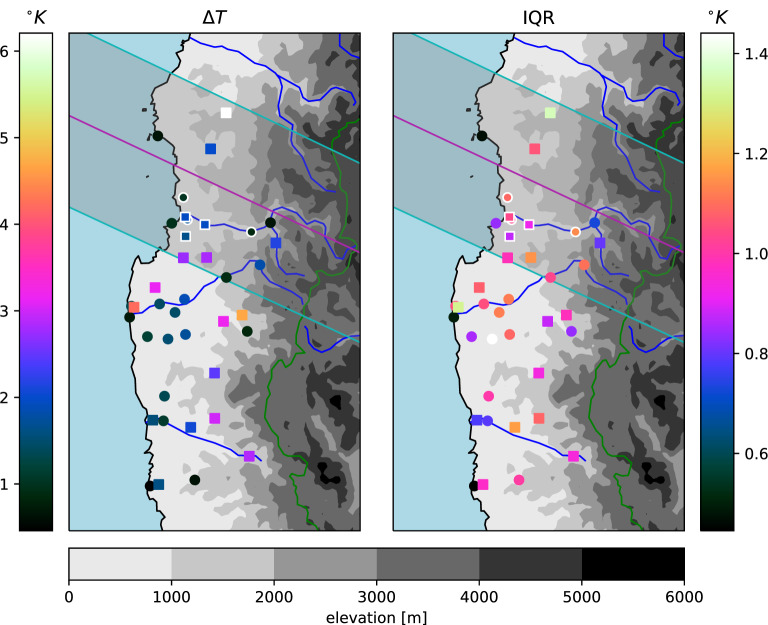
Estimated maximum 2m air temperature depressions ($\Delta T$) during the eclipse (left), based on the smoothing type lasso regression, and IQR of the estimates (right). The lasso estimates for $\Delta T$ are corrected for the bias of the error distribution. Square symbols mark estimates which fall outside 95% of the test error distribution for a given station, and white marker edges correspond to the stations shown in Figs. 1 and 2. Cyan lines demarcate the edges of the band of totality and the magenta line its center^61^. Gray shading indicates terrain elevation^62^.

**Figure 4:**
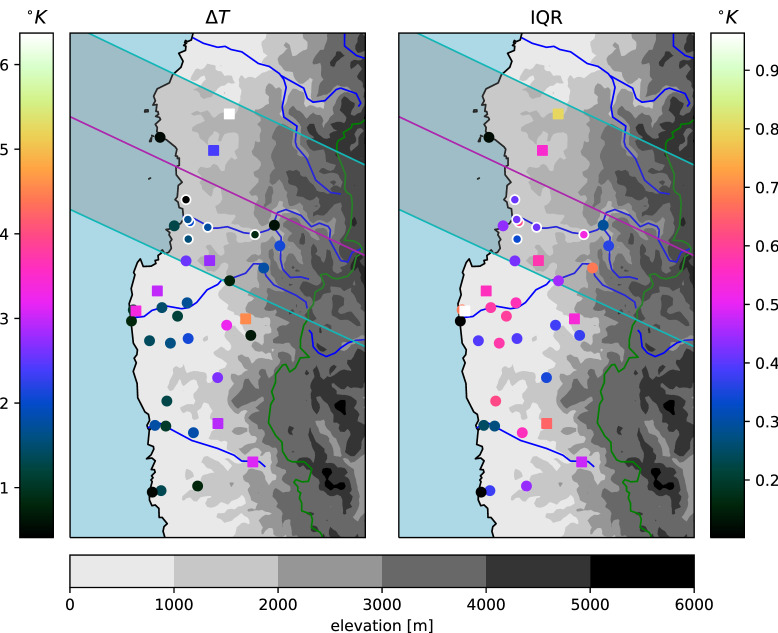
Estimated maximum 2m air temperature depressions as in Fig. 3 (symbols as described there), but for estimates based on the automatic relevance determination (ARD) regression. IQR here is 0.67 times the standard deviation of the predictive distribution.

**Figure 5:**
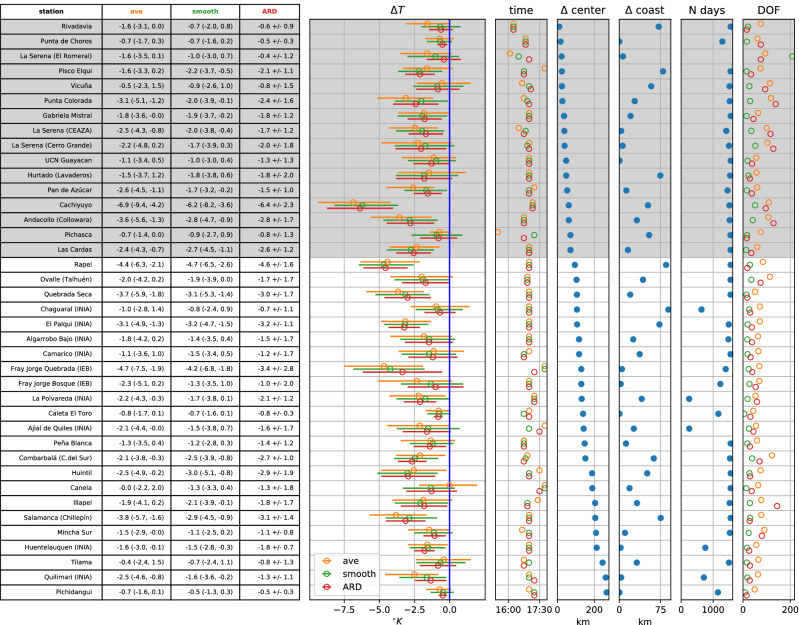
$\Delta T$ estimates for stations in the Coquimbo Region with at least 1 year of available data at a resolution of at least 15 min. For lasso regressions (average and smoothing), uncertainties are given as 2.5 and 97.5 percentiles of the test errors separately, while for ARD regression, one value of 1.96 times the standard deviation of the predictive distribution is given. Lasso estimates are corrected for the bias of the error distributions. Errorbars on plots correspond to these ranges. ‘Time’ is the time of maximum $\Delta T$ for each type of estimate; ‘$\Delta$ center’ is the distance from a station to the eclipse’s center line; ‘$\Delta$ coast’ the distance to the coast. ‘N days’ is the number of available days on record, and ‘DOF’ are the degrees of freedom (number of regression coefficients) for each estimate.

Besides the empirical estimates for uncertainties, we also employ a Bayesian regression technique which estimates a full posterior distribution. Certain specific regularisation penalties in classical regression correspond to particular distributions of priors over the parameters in a Bayesian setting^54^, and we chose automatic relevance determination (ARD)^55^ as a method that selects predictors based on the posterior distributions of their associated coefficients. The agreement between the estimates and their uncertainties obtained from the three methods (Figs. 3, 4, 5) is encouraging. Note that a Bayesian regression would be meaningless in the ‘average’ direction, since the time range of interest $t_{\bullet }$ is not actually the target in that case.

Stronger temperature depressions due to the eclipse are generally found further inland (Figs. 3, 4), which is expected due to the thermal inertia of the sea. Only one station close to the coast shows a larger $\Delta T$ estimate (Fray Jorge Quebrada)—but it should be noted that the timing of the largest temperature drop is late there compared to most stations (Fig. 5), suggesting it may have been related to the sunset rather than the eclipse (the station is separated from the sea and setting sun by a mountain range). There is a hint of a suggestion that moving away from the center line of the eclipse reduces the experienced temperature drop, especially outside of the region of totality, but the proximity to the coast appears dominant. The largest estimated temperature drop of more than $6^{\circ }$ C (Cachiyuyo) might raise suspicions, but it turns out that its magnitude is largely a result of its occurrence at the end of the day, when temperatures are dropping very fast even on regular days and the eclipse’s effect is more akin to an earlier onset of nightfall (cf. Fig. 7).

### The eclipse wind

Encouraged by the results for the surface temperatures, we apply the smoothing type lasso also to the wind field. The average type regression gives quantitatively very similar results but is both much more costly computationally (at least if uncertainties are to be estimated) and harder to analyse since its setup differs from regular regression frameworks in that the information of interest (times $t_{\bullet }$) are not actually the regression’s target. The results (not shown) with respect to uncertainties are very similar to those for the temperature case, with some but not all stations achieving a statistical significance of the estimated eclipse effect at 95%; we performed the ARD variant of the regression as well with comparable results.

**Figure 6:**
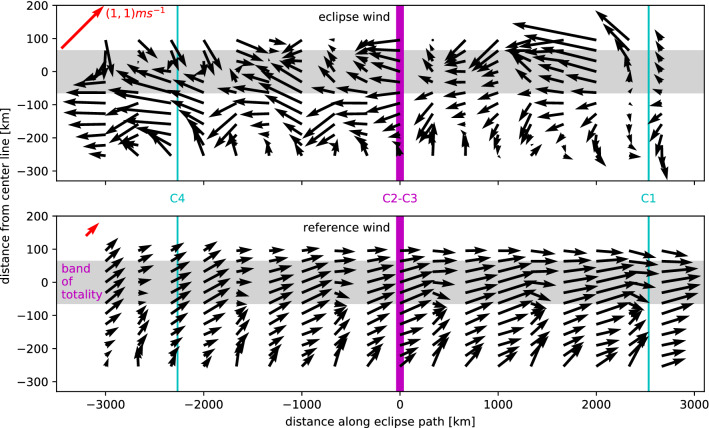
Vector wind as a function of distance along (x) the path of the eclipse and from the center line (y). Eclipse wind is the observed wind vector minus the reference estimate. The x distance is calculated from observation time assuming an umbral velocity of 2000 km h$^{-1}$; the positive direction points west to east as usual since the eclipse’s movement is also in this sense. The field is smoothed with biquadratic radial basis functions. While the x, y *locations* are non-square, the arrow lengths are isotropic; red arrows show the scale of the wind with u = v = 1 ms$^{-1}$.

Instead of focusing on uncertainties as in the temperature case, we simply consider the point estimates for the change in the wind field induced by the eclipse. We applied the regression to the u, v wind vectors separately and calculate an ‘eclipse wind’ following Clayton^3^ by subtracting the reference estimate from the observations on eclipse day. A two-dimensional spatial picture can be constructed, again following Clayton, by plotting a station’s relative latitudinal distance to the center path of the eclipse in y, and replacing the time of an observation by a virtual distance along the eclipse’s path calculated with the assumption of a propagation speed of the eclipse of approximately 2000 km h$^{-1}$ (Fig. 6).

Clayton hypothesised that an eclipse provided an optimal occasion for the assessment of earlier theories by Ferrel regarding the structure of cyclones with a cold core^56^. The essence of the argument is that a cool surface temperature anomaly will lead to surface outflow of air and replacement by subsiding motion, with ensuing inflow aloft and possibly a closing of the vertical cell at some distance from the center. Outflow is associated with anticyclonic and inflow with cyclonic rotation, but air which has acquired cyclonic angular momentum aloft will conserve it when subsiding. The outflow therefore has to first overcome the existing cyclonic momentum before crossing a circle of zero rotation and turning anticyclonic at some distance from the center of subsidence. Clayton surmised, however, that because of friction, the cyclonic rotation of the innermost ring would be too weak to be observable, and focused on demonstrating an anticyclonic sense of rotation further away.

**Figure 7:**
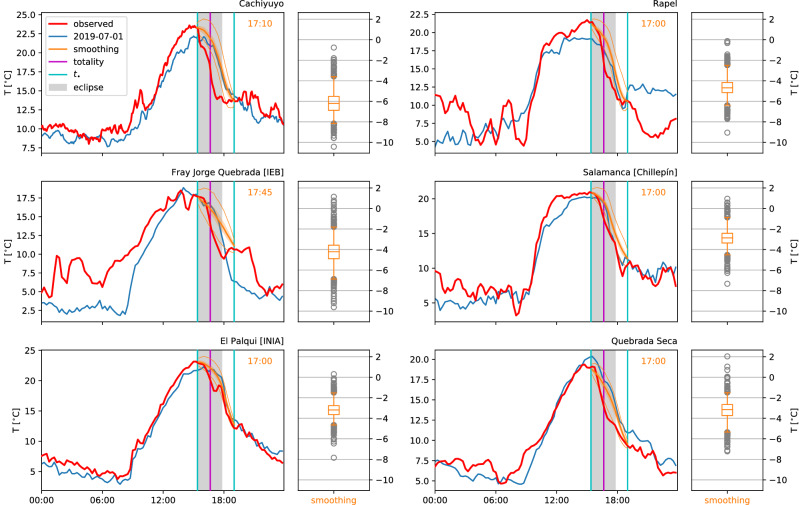
Observed 2 m air temperatures and smoothing lasso reference estimates for the day of the eclipse as in Fig. 1, but for the stations with the largest $\Delta T$ estimates. Additionally, the observed temperatures during July 1st, 2019 (the day preceding the eclipse) are shown, to give an idea of the realism of the estimates.

The wind field is very variable and a result of many interacting forces, including local to continental-scale contrasts in surface heat fluxes and the effects of synoptic weather systems. The signal-to-noise ratio of any eclipse-induced effects may be rather low, and Clayton’s results have not been reproduced convincingly^2,5^. Instead, where changes have been demonstrated, explanations other than his proposed ‘cyclone’ have been favoured^4^. It should also be noted that Ferrel’s and Clayton’s ideas about cyclones predate the modern understanding of midlatitude and tropical cyclones as primarily the result of dynamical instabilities by half a century.

A noteworthy feature of the eclipse wind in Fig. 6 is the ‘bow shock’ on the eastern (C1) front, where air seems to move out of the way of the arriving eclipse. Since the eclipse travels with supersonic speed, it is impossible for any eclipse-caused outflow to result in air parcels moving ahead (eastward) of the eclipse. Remember, however, that while the frame of reference for the wind vector *locations* in Fig. 6 can be considered to be moving with the eclipse, the same is not true for the coordinate system of the quivers, which is both isotropic and static. The fact that eastward flow occurs at some stage during the eclipse is therefore not a contradiction. (As an aside, the strong anisotropy of the virtual along-path direction related to the eclipse’s supersonic movement casts doubt on Clayton’s original argument and his Fig. 4, which represents the eclipse cyclone as a fairly symmetric structure.) With much imagination, one might discern an anticyclonic movement of the air masses following C1, which later turns into a cyclonic movement around the time of totality. This would imply an initial anticyclonic outflow, followed by subsidence which carries with it a cyclonic vorticity stemming from the required inflow aloft. It could further be argued that the flow field appears more turbulent after the passing of totality than before, analogously to that in the wake of a fast-moving solid object.

Without more detailed analysis, which is beyond the scope of this study, we cannot confirm or refute any of the preceeding interpretations; and others are equally possible: for example, it appears that zones of converging and diverging flow alternate in the penumbral zone, with convergence on the northern edge coexisting with divergence to the south and vice versa. Similarly strong gradients in surface heating as across the edge of the umbra may be found over ocean eddies^57^ and fronts^58,59^, resulting in wind field adjustments which could be compared to the eclipse case. However, the supersonic movement of the umbral shadow is likely to result in a very different dynamical adjustment of the atmosphere.

In the Coquimbo Region, the wind field is influenced by the Southeast Pacific Subtropical Anticyclone, passing frontal systems, a strong land-sea breeze and equally strong orographically driven wind systems. Around La Serena, both the land-sea and the mountain-valley systems tend to produce westerly winds during the day and easterlies at night, but differences in reversal timing may lead to complex interactions. The eclipse occurred just before sunset, and an earlier-than-usual reversal of the daily wind systems due to the associated decreased surface heating is consistent with the mostly easterly direction of the eclipse wind in Fig. 6.

## Discussion

After witnessing the eclipse in La Serena on July 2nd, 2019, we were interested in finding out how much the atmosphere’s surface layer had cooled during the event. Observers reported that they felt colder during the eclipse than before, but that does not necessarily imply that the air temperature dropped considerably, since much of the sensation may be due to an experienced reduction in direct radiative heating of the body. CEAZAMet operates a monitoring network with many stations inside the belt of totality of this eclipse which provided us with near-surface air temperature data. However, no ‘obvious’ pattern of change, such as a pronounced dip in the temperature curve, is consistently discernible at all or a majority of the stations. The eclipse occurred so close to sunset that temperatures had started dropping before C1 and little radiative energy was available after C4 to raise temperatures again. Furthermore, any features of the data from the day of the eclipse which could potentially be interpreted as dips in temperature do not stand out against the general background of intra-diurnal variability in the Coquimbo Region. This is because the dominantly southerly wind alternates frequently between a slight on- and offshore flow, which brings with it strong advective temperature changes since a cold ocean borders a strongly heated land surface.

We therefore chose to focus on the problem of how to separate the effect of the eclipse from other circumstantial influences on the observed meteorological variables. The reference estimate of how a meteorological variable would have behaved on the same day but in absence of the eclipse is speculative, and we cannot confidently use its point value without some idea of how uncertain it is. While some previous studies give confidence intervals on certain specific estimates^4,5^, these are not related to the uncertainties surrounding the actual effect sizes at specific times and locations. Here, we calculate uncertainties and their bias (for the lasso estimates) empirically on the basis of longer-term records from each of the stations at which we observed the eclipse, by performing our estimation procedure for any non-eclipse day and calculating the errors with respect to the true evolution of that day. The error distribution thus pertains to any arbitrary day of the year and includes uncertainties arising from annual-scale variability; in areas with a more pronounced annual cycle than the Coquimbo region, we would expect the estimates to exhibit larger uncertainties, which in turn could be counteracted by restricting the training data set to days closer to the day to be predicted (in terms of the day of year, DOY) if longer-term records are available.

Calculating the errors associated with particular estimates allows for selecting one with a low uncertainty. The lowest test errors are achieved by the smoothing lasso and the ARD regression (Table 2), but the error for ARD is taken to be simply the posterior standard deviation and may not be entirely accurate since ARD assumes the data to be Gaussian-distributed. It is convenient that the smoothing-type estimates perform the best, since they are both more time-efficient than the average-lasso and take the form of a regular regression. Therefore different regression methods can be used interchangeably and analysed with well-established tools such as cross-validation. All the methods we have described possess an adjustable parameter which we select to approximately minimise a test error; for the lasso regressions it is the regularisation parameter $\alpha$, for the unweighted average it is the number of days $N$, and for the local regressions it is the kernel width. The estimates are not overly sensitive to the parameter values, and a complete numerical optimization over all stations is computationally expensive for some methods. Additionally, different stations’ estimates have rather different error levels and a general loss function would need to be able to weight those appropriately. Therefore, we simply carry out a grid search for a selection of stations and choose roughly appropriate parameter values.

**Table 2:** Test errors over all stations and all non-eclipse days for the methods discussed in this paper. Since we perform ARD only for a subset of times out of $t_{\bullet }$, we restrict all errors to this subset. The ARD error is the posterior standard deviation averaged over times and stations. The other errors are computed to be comparable with this, as the RMSE over days in $d_{\circ }$, averaged over times and stations.

Method	Err
Unweighted average	1.15
Linear local regression	1.18
Quadratic local regression	1.0
‘Average’ lasso	0.9
‘Smoothing’ lasso	0.8
‘Smoothing’ ARD	0.61

It is conceivable that methods with better performance can be found. We limit ourselves to predicting diurnal temperature cycles by using other diurnal temperature cycles as training data. A regression framework is more typically used to relate one variable to another, and this could certainly be done here; however, contemporaneous multi-variable regressions cannot be used since additional independent variables may also be affected by the eclipse. To the extent that the values of other variables may be able to differentiate locations in state space characterised by very similar temperatures, it could be useful to include them. Similarly, it might be possible to exploit autocorrelations on an inter-diurnal scale. We experimented with non-linear regression methods (Gaussian Processes, Neural Networks) for the more appropriate smoothing setup but found no improvements, which is explained by the dominant influence of only the two data points immediately before and after the prediction period $t_{\bullet }$. We also experimented with using the solar elevation angle $\beta$ as a coordinate instead of time, since it may be expected that it normalise annual variability and guard against the impact of varying day lengths to some extent, but similarly found no improvement to the predictive accuracy of our models while significantly complicating the analysis.

A physics-based model could in principle be initialized to the state of the atmosphere at the start of the eclipse and run predictively with eclipse-unaware radiative forcing in order to provide a reference estimate. If all necessary physical parameters are measured for a specific location, this could be a relatively confined boundary layer energy balance model. However, in our case we have little knowledge of important parameters such as surface albedo, boundary layer thickness and longwave radiation. As we have previously stated, advection plays an important role in the surface energy balance, and either a crude observational estimate based on widely spaced station measurements or a full numerical weather prediction (NWP) model is needed in order to account for it. However, such a physics-based modeling exercise represents a major effort, and we devised our statistical methods primarily in order to provide a simpler alternative. It is furthermore not clear whether the results from a physical model would be less uncertain than those of a statistical one, in particular under the circumstances described for the Coquimbo Region: relatively small swings between on- and offshore winds can have major effects on local temperatures, and the horizontal resolution of NWP models is coarse compared to the complexities of the terrain. Even more importantly, adequate data for model initialization is not available.

## Methods

In terms of the data matrix $X$ with rows corresponding to days and columns to times, the ‘average’ type regression estimate for $d_{\bullet }$ is given by

$$ \hat{Y}_{d_{\bullet }t} = X^T_{d_{\circ }t} \hat{\beta }_{d_{\bullet }t_{\circ }} $$

where $\hat{\beta }$ is an estimate for the (column) vector of regression coefficients $\beta$ and $\hat{Y}$ is the regression estimate. This expression corresponds to an unweighted average over $N$ (selected) days if $\beta$ contains $N$ elements with value $1/N$ and otherwise zeros. Viewed as a regression, an intercept is included by adding a column of ones to $X^T$, and the estimate $\hat{\beta }_{d_{\bullet }t_{\circ }}$ for $\beta$ is obtained by minimising a loss function. We denote with the subscripts the fact that $\hat{\beta }$ is estimated by using the observation at $d_{\bullet }$ but only the times $t_{\circ }$ (excluding the eclipse) as a target.

The ‘smoothing’ approach consists in using the columns of $X$ corresponding to $t_{\bullet }$ as the regression’s targets and all remaining columns $X_{dt_{\circ }}$ as predictors:

$$ \hat{Y}_{dt_{\bullet }} = X_{dt_{\circ }} \hat{\beta }_{d_{\circ }t_{\bullet }}. $$

In this case, all non-eclipse days $d_{\circ }$ on record serve as training samples, and estimates are obtained for any day but only for $t_{\bullet }$. The regression coefficients $\beta$ in this case correspond to weights of a smoothing kernel applied to the day of the eclipse, where the values at $t_{\bullet }$ have been removed from and filled in by the smoothing procedure.

We calculate a distribution over prediction errors by subtracting the observed value at $t_{\bullet }$ for all non-eclipse days from the corresponding estimates:

$$ PE = \hat{Y}_{d_{\circ }t_{\bullet }} - X_{d_{\circ }t_{\bullet }}. $$

In the ‘average’ type approach, the regression’s target are the observations $X_{d_{\bullet }t_{\circ }}$ and information from $t_{\bullet }$ never enters the calculations. We can hence think of all observation-prediction pairs for the eclipse as a test set and compute statistics on $PE$ in the same way as we would on the observations themselves.

The ‘smoothing’ type regression is a standard setup where targets $X_{d_{\circ }t_{\bullet }}$ and estimates $\hat{Y}_{d_{\circ }t_{\bullet }}$ are paired, and we can use cross-validation to divide the date dimension $d$ into training and test sets. Since we operate with approximately 4 years of data, we use 4-fold cross-validation, such that the test sets consist of roughly a full year of data. The distribution of $PE$ is taken to be the set of its values for all days $d_{\circ }$ calculated whenever a given day is in a test set. The final estimate of the variable’s eclipse-unaffected value at the day of the eclipse is calculated from all days $d_{\circ }$.

The lasso, which we apply to both regression problems, is a regularised regression whose loss function contains a $\ell ^1$ penalty on the vector of regression coefficients $\beta$. It is this form of the penalty that leads to the coefficients for less influential predictors to be set to zero, and thereby to subset selection^54^. The penalty is scaled by a hyperparameter, denoted $\alpha$ here, which controls how many predictors are culled: the larger the value of $\alpha$, the more coefficients become zero, and the lower the resulting model complexity. We choose its value by approximately minimising a root mean square test error (RMSE) computed by averaging $PE^2$ over $t_{\bullet }$ and $d_{\circ }$. The regression estimate itself is not overly sensitive to the precise value of $\alpha$, and so we simply choose a single value that is approximately optimal for a number of meteorological stations (see Table 3).

**Table 3:** Values and types of the hyperparameters for the different estimation methods described.

Method	Hyperparameter	T2	u, v
Unweighted average	Number of days	10	
‘Average’ lasso	$\alpha$	0.005	
Local linear/quadratic regression	Length scale (min)	50	
‘Smoothing’ lasso	$\alpha$	0.1	0.05
‘Smoothing’ ARD	$\lambda$ Threshold	$10^4$	$10^4$

We compare the lasso estimates with estimates based on simple averaging and smoothing. For the averaging, the data matrix’s date dimension is first ordered according to the mean square difference over times $t_{\circ }$ between each day and eclipse day. Then, the $N$ closest days, in this Euclidean distance sense, are averaged. A lower value of $N$ corresponds to a larger value of $\alpha$ (stronger regularisation and fewer non-zero coefficients). As with $\alpha$, we select a value for $N$ which approximately minimizes the average test error over all stations.

For smoothing, we use local linear and quadratic regression with a Gaussian kernel^54^. The period $t_{\bullet }$ is removed before the smoothing estimate is computed and the resulting gap filled in by the estimate. The governing parameter for local regressions is a length scale, which is again fit by approximately minimizing the average test error over all stations.

An additional regression type, linear Bayesian regression with automatic relevance determination (ARD), is applied to the smoothing problem. The prediction error is here estimated in the form of a full predictive distribution rather than empirically for a point estimate. Since the eclipse period $t_{\bullet }$ is what we want to compute any errors for and the average type regression does not use it as a target, the Baysian regression does not really provide any useful information in this case. ARD regularises complexity by pruning predictors whose distributions’ precision $\lambda$ (the inverse of the standard deviation) exceeds a threshold value^55^. In contrast to the lasso, however, the numerical value of this threshold has almost no effect on the results and can be left at a default setting (see Table 3). Note, however, that the implementation used^60^ supposes Gaussian distributions for both the data and the regression coefficients (and therefore also the predictive error distribution), an assumption that is only approximately valid in our case.

## Data Availability

The datasets generated and analysed during the current study are available from the corresponding author on reasonable request.
